# Long‐read transcript sequencing identifies differential isoform expression in the entorhinal cortex in a transgenic model of tau pathology

**DOI:** 10.1002/alz.089436

**Published:** 2025-01-03

**Authors:** Szi Kay Leung, Aaron Jeffries, Isabel Castanho, Rosemary Bamford, Emma Dempster, Zeshan Ahmed, Eilis Hannon, Jonathan Mill

**Affiliations:** ^1^ University of Exeter, Exeter United Kingdom; ^2^ Harvard University, Boston, MA USA; ^3^ University of Exeter, Exeter, Devon United Kingdom; ^4^ Eli Lilly & Co Ltd, Windlesham United Kingdom

## Abstract

**Background:**

Increasing evidence suggests that alternative splicing plays an important role in Alzheimer’s disease (AD), a devastating neurodegenerative disorder involving the intracellular aggregation of hyperphosphorylated tau.

**Method:**

We used whole transcriptome and targeted long‐read cDNA sequencing to profile transcript diversity in the entorhinal cortex of wild‐type (WT) and transgenic (TG) mice harbouring a mutant form of human tau.

**Result:**

Whole transcriptome profiling showed that previously reported gene‐level expression differences between WT and TG mice reflect changes in the abundance of specific transcripts. Ultradeep targeted long‐read cDNA sequencing of genes implicated in AD revealed hundreds of novel isoforms and identified specific transcripts associated with the development of tau pathology.

**Conclusion:**

Our results highlight the importance of differential transcript usage, even in the absence of gene‐level expression alterations, as a mechanism underpinning gene regulation in the development of neuropathology. Our transcript annotations and a novel informatics pipeline for the analysis of long‐read transcript sequencing data are provided as a resource to the community.

